# Times of Change – “Eppur si muove!” or “Parturient montes, nascetur ridiculus mus”?

**DOI:** 10.1007/s40319-022-01251-1

**Published:** 2022-09-12

**Authors:** Christine Godt

**Affiliations:** grid.5560.6 0000 0001 1009 3608 Dr.; Professor of European and International Economic Law, Carl von Ossietzky University, Oldenburg, Germany

On 17 June 2022, the Ministerial Conference (MC) of the World Trade Organization (WTO) adopted a so-called “waiver decision” on COVID-19 vaccines. Other than clarifying TRIPS flexibilities, it waives quantitative restrictions on import and export under Art. 31f TRIPS. It does not install a full-fledged COVID-related *patent* waiver as applied for by South Africa and India on 2 October 2020, which became so heavily contested. How should this newest TRIPS Council decision be evaluated? After the stalemate of the WTO in recent years, does this decision signal to the world that the multilateral system is still functioning and the WTO is able to produce compromise (“*Eppur si muove*”^1^)? Or is this decision so minuscule that it is not worth an editorial (“*Parturient montes, nascetur ridiculus mus*”^2^)?

A closer look reveals a mixed picture. After a lengthy and contested debate in all (including academic^3^) fora, the adopted decision (WT/MIN(22)/30) neither follows the text of the 2020 patent waiver application, nor is the text identical to the WTO Secretariat’s submissions of 3 May or 10 June 2022, nor will this text mark the end of the discussion. On 6 July 2022, the TRIPS Council welcomed the MC decision and announced further deliberations on extensions to COVID-19 diagnostics and therapeutics.

The waiver remedies the inefficiencies of Art. 31f TRIPS, which admittedly contradict WTO’s own principles.^4^ In this, it echoes economists’ scepticism on product export restrictions which prevent economies of scale, disturb supply chains and in fact reduce the overall availability of products. Yet, it is path-dependent. It narrowly limits the scope of import/export permits to developing countries without manufacturing capacities, it is limited to vaccines, and is redundant on existing flexibilities.^5^

Thus, it does not enable technology transfer under Art. 66 TRIPS. This is the most important fall-back. In contrast to the AIDS crisis at the end of the 1990s, the core of the COVID-19 crisis has less been about the supply of products but more about the containment of the virus. The latter requires continuous R&D. The idea was to build up personal and institutional capacities to develop domestic emergency responses and thus enhance the world’s likeliness to limit the expansion of future mutations. In the course of 2021, it became evident that many developing countries are potentially able to produce qualitatively high-level vaccines (in contrast to prejudices and lobbyists’ narratives). The Institute Pasteur maintains several subsidiaries on the African continent – not to mention India and Pakistan as major producers. Yet, the WTO Secretariat’s (bracketed) technology-oriented proposal as submitted to the TRIPS Council by the negotiating group on 3 May 2022^6^ did not survive the MC’s deliberations. It foresaw a clause for WIPO to assist eligible countries, along with a window for amendments to include further patents.

This (now missing) bracketed text language sheds light on the central failure of the last two years. Since the beginning of the pandemic, industrialised countries prioritised production over technology transfer. It is for this reason that the WHO-CoVax initiative failed,^7^ state subventions were not conditioned,^8^ and activities to build-up production *in Africa* remained embarrassingly limited.^9^ The emphasis was to retain proprietary control over technologies which are conceived as the technological basis for future therapies, not only virus infections but also cancer. Even the sharing initiative by the US administration to provide the UN-backed COVID-19 Technology Access Pool (C-TAP) and its Medicines Patent Pool (MPP) with access to 11 NIH technologies was aimed to accelerate generic production and *then* supply to mid and low-income countries. While the tone changed with the US Biden administration taking office in January 2021, the EU’s policy remained characterised by a clear opposition to a patent waiver coupled with a commitment to CoVax. Yet, the CoVax initiative is no success story. It was undermined by its proponents (donor countries and manufacturers).^10^ In 2021, CoVax did not deliver enough doses; in mid-2022, CoVax refused further donations because of oversupply.^11^ Since the winter of 2021/2022, millions of doses (of both AstraZeneca and Moderna) have been destroyed after having exceeded the expiration date. Yet, what is needed is a technology transfer approach which helps to respond to emergencies domestically and promptly. In correlation to the passing of time, vaccination scepticism also grew in Africa and the policy of product donations came to be perceived as a further instrument of colonialisation. Some argued that technology transfer is not the core business of private companies, and that the patent waiver would not have remedied the vaccine shortage; no refusal to license became known. Yet, a broader TRIPS waiver, as an instrument of public international law, would have signalled to the WTO states that industrialised countries support technology transfer. Under a TRIPS-waiver regime, domestic compulsory licenses still had to be issued under national law. But in fact, the dominant product-oriented policy undermined initiatives for compulsory licenses. Not one single compulsory license was ever issued. The global problem is evident: the world is missing out on opportunities to promptly react to mutations.

Despite this criticism, the MC’s decision will not remain without effects. It signals a renewed ability for consent in WTO decision-making (in contrast to more narrow constellations, such as the G7 or EU, or broader ones, such as the UN). In this, it showcases that specialised multilateral institutions hold advantages over other fora, such as territorially defined regional blocks or bilateral negotiations. Today, this is an important signal. In addition, the decision sheds light on the WTO’s institutional development in two aspects: First, the decision became possible through a new type of horizontal collaboration between the WTO, WHO and WIPO. While CoVax melted down to become an instrument of mercy, and WIPO’s assistance support was cut out during the MC’s negotiations, the developments in 2022 shed light on institutional change. The WTO, WHO and WIPO cooperated productively. They formed C-TAP under the WHO, a trilateral Technical Assistance Platform under WIPO, and initiated a Pandemic Prevention Fund under the auspices of the World Bank with the technical expert control of the WHO. This mirrors the emergence of a modern regime-complex where intergovernmental institutions collaborate productively directly with each other (and not via diplomatic channels). It is against the background of the waiver debate, that innovative licensing initiatives such as the MPP under C-TAP (WHO) are put in the spotlight. While the TRIPS patent waiver did not achieve consensus, the NIH did waive central patents and “gave” them to the MPP for further administration. This shows that “patent donations” require both political decision-making and governmental engagement. Second, we are witnessing a shift towards different actors. Since March 2021, the Nigerian-born Ngozi Okonjo-Iweala is the WTO Director-General, and Ambassador Lansana Gberie of Sierra Leone is the Chairperson of the TRIPS Council. Under their leadership, a slim “нeт” was no longer possible, and paved the way to the current waiver version. The basic June decision was finetuned by further alliances and commitments.^12^

All in all, fundamental transformations continue to change our lives. Digitalisation, climate change, the Covid-pandemic and the war on Ukraine territory, all account for profound transformations in international organisations and a new world order with strong aspirations to more national sovereignty. The latest G7 summit on 28 June 2022 mirrored these changes. The countries were unable to come up with a joint final declaration; threshold countries did not join the alliance of the West against Russia, and even met in advance to consult with China and Russia. Against this background, the novel collaborative institutional arrangement in the WTO appears as a blessing. It affects the foundations of the economic order such as property and contract and the competitive order in order to re-calibrate the relationship between rights and duties, freedoms and obligations. There is reason for hope.

## Footnotes

1 English “And yet it moves”. Attributed to Galileo Galilei, who uttered the phrase in 1633 after being forced to recant his claims that the earth moves around the sun. Stephen Hawking (2003) *On the Shoulders of Giants: The Great Works of Physics and Astronomy*, Running Press: Philadelphia, PA., pp. 396–397.

2 Horace, *Ars Poetica*, p. 139, (English “The [roaring] mountain just gave birth to a mouse!”).

3 *See* the (waiver-opposing) Position Statement of the Max Planck Institute for Innovation and Competition of 7 May 2021 (supported by a number of academics and practitioners: https://www.ip.mpg.de/de/forschung/meldungen-aus-der-forschung/covid-19-and-the-role-of-intellectual-property-list-of-supporters.html) in comparison to the (waiver-supporting) open letter of many renowned academics of July 2021 (http://infojustice.org/archives/43313).

4 Pondered early from a jurist’s perspective: Joost Pauwelyn (2020) “Export Restrictions in Times of Pandemic: Options and Limits under International Trade Agreements”, 54:5 Journal of World Trade pp. 727–741; empirically pinpointed by economists: Chad P. Bown and Thomas J. Bollyky (Aug. 2021) “How COVID-19 vaccine supply chains emerged in the midst of a pandemic”, Peterson Institute for International Economics-Working Paper 21-12; from an NGO perspective: James Love (2022) “The June 17, 2022 WTO Ministerial Decision on the TRIPS Agreement, Knowledge Ecology International, Post of June 17, 2022”, (https://www.keionline.org/3783).

5 As expressed by many commentators, *inter alia* the MPI Position Statement of 5 July 2022 on the Decision of the WTO ministerial Conference on the TRIPS Agreement adopted on 17 June 2022 (https://www.ip.mpg.de/en/research/research-news/position-statement-on-the-decision-of-the-wto-ministerial-conference-on-the-trips-agreement.html), and James Love (*supra* note 4).

6 *See* para. 3(a) Waiver Proposal Version of 3 May 2022 (IP/C/W/688).

7 Antoine de Bengy Puyvallée and Katerini Tagmatarchi Storeng (2022) “COVAX, vaccine donations and the politics of global vaccine inequity”, Globalization and Health 18:26 (https://doi.org/10.1186/s12992-022-00801-z).

8 The German government engaged heavily e.g. in the Biotech Start-up CureVac in 2020. Prompted by rumours about a planned take-over by the US Trump administration, the German government first bought shares in March 2020 (EUR 300 million, budgeted to the ministry of economy). Similar to German Lufthansa subventions in 2020, but in contrast to other European countries (e.g. the Netherlands tied subsidies for KLM to an engagement in green H2-engine technologies), no conditions were attached, such as equitable licenses or tiered pricing. Later in September 2020, the German government invested a further EUR 252 million into an acceleration of vaccines production, budgeted to the ministry of research (Lars Petersen, buisinessinsider.de, 6 September 2020).

9 BioNTech’s “BioNTainers”, Press Release by BioNTech of 23 June 2022; its first production site will be Kigali (Ruanda).

10 De Bengy Puyvallée and Storeng (2022), *supra* note 7.

11 *Frankfurter Allgemeine Zeitung*, 19 July 2022, p. 1.

12 Joint presidential declaration of 15 August 2022 by Paul Kagame, President of Rwanda; Emmanuel Macron, President of France; Cyril Ramaphosa, President of South-Africa; Macky Sall, President of Senegal; Olaf Scholz, Chancellor of Germany; Tedros Ghebreyesus, General Secretary WHO; *Frankfurter Allgemeine Zeitung*, 16 August 2022, p. 8.

